# Automated estimation of cancer cell deformability with machine learning and acoustic trapping

**DOI:** 10.1038/s41598-022-10882-w

**Published:** 2022-04-27

**Authors:** O-Joun Lee, Hae Gyun Lim, K. Kirk Shung, Jin-Taek Kim, Hyung Ham Kim

**Affiliations:** 1 Department of Artificial Intelligence, The Catholic University of Korea, Bucheon, 14662 Republic of Korea; 2 Department of Biomedical Engineering, Pukyong National University, Busan, 48513 Republic of Korea; 3 Department of Biomedical Engineering, University of Southern California, Los Angeles, CA 90089 USA; 4 Department of Convergence IT Engineering, Pohang University of Science and Technology, Pohang, 37673 Republic of Korea

**Keywords:** Biomedical engineering, Cancer screening

## Abstract

Cell deformability is a useful feature for diagnosing various diseases (e.g., the invasiveness of cancer cells). Existing methods commonly inflict pressure on cells and observe changes in cell areas, diameters, or thickness according to the degree of pressure. Then, the Young’s moduli (i.e., a measure of deformability) of cells are estimated based on the assumption that the degrees of the changes are inversely proportional to Young’s moduli. However, manual measurements of the physical changes in cells are labor-intensive, and the subjectivity of the operators can intervene during this step, thereby causing considerable uncertainty. Further, because the shapes of cells are nonuniform, we cannot ensure the assumption for linear correlations of physical changes in cells with their deformability. Therefore, this study aims at measuring non-linear elastic moduli of live cells (degrees of cell deformability) automatically by employing conventional neural networks (CNN) and multilayer perceptrons (MLP) while preserving (or enhancing) the accuracy of the manual methods. First, we obtain photomicrographs of cells on multiple pressure levels using single-beam acoustic tweezers, and then, we suggest an image preprocessing method for emphasizing changes in cell areas on the photomicrographs. The CNN model is trained to measure the ratios of the cell area change at each pressure level. Then, we apply the multilayer perceptron (MLP) to learn the correlations of the cell area change ratios according to the pressure levels with cell deformability. The accuracy of the CNN was evaluated using two types of breast cancer cells: MDA-MB-231 (invasive) and MCF-7 (noninvasive). The MLP was assessed using five different beads (Young’s moduli from 0.214 to 9.235 kPa), which provides standardized reference data of the non-linear elastic moduli of live cells. Finally, we validated the practicality of the proposed system by examining whether the non-linear elastic moduli estimated by the proposed system can distinguish invasive breast cancer cells from noninvasive ones.

## Introduction

Since the mechanical stiffness of breast cancer cells represents their invasion potential and future metastasis, various biophysical techniques have been developed to estimate the Young’s modulus (*E*) of a single cell^1–5^. Atomic force microscopy (AFM) is a widely used method for measuring the mechanical properties of cells^4,6–8^. However, the intrinsic limitation of AFM—the direct contact between the cantilever and the cell—can cause physical damage such as the destruction of the cell membrane^9^. Other disadvantages include the need for a holding tool to fix the suspended cell in one place, and the lateral instability of suspended cells that exists when measuring with cantilevers^10^. These challenging factors unfortunately lead to a high margin of error^11–14^.

The deformability of cancer cells has also been measured using optical tweezers^15–17^. Optical tweezers invented by Ashkin^18,19^ have been widely used to manipulate atoms and nanoparticles for studying cellular properties^20–22^ and DNA-protein complexes^23,24^ owing to its advantages of being nanometer-scale^25–27^. However, a laser power of 800 mW can only generate the trapping force in the range of piconewtons, which limits their application^28^. Further, two particles need to be attached to the opposite side of the cell because optical tweezers cannot directly trap the cell in the general optical setup^29,30^. In this case, controlling the binding sites of the particles to the cell membrane is a challenging process^15^.

High-frequency ultrasound microbeam is an emerging technique for biomedical research that has the advantages of noncontact and strong radiation pressure^31–33^. A single-beam acoustic tweezer (SBAT) uses a tightly forced ultrasound microbeam to manipulate the cell or microparticle for single-cell analysis and for measuring intercellular properties^34–37^. Since the high-frequency SBAT can generate the trapping force in the range of piconewtons to nanonewtons^38–40^ and radiation pressure up to a few megapascals, this tool is a good alternative for directly trapping and deforming suspended cells to quantify cell mechanics. Recently, SBAT was shown to be feasible for measuring cell deformability (Young’s modulus) qualitatively or quantitatively without damaging the cell surface^41–43^.

However, this method has a few limitations. First, the existing studies assumed that changes in cell areas on the photomicrographs are directly proportional to the pressures inflicted by the SBAT. Thus, Lim et al.^41^ calculated the average gradient of the area change ratios according to pressure. Further, they assumed that the ratio is inversely proportional to *E*. Finally, this method manually measures the area change ratios of cells by drawing cell boundaries on photomicrographs and counting pixels within the boundaries.

**Figure 1:**
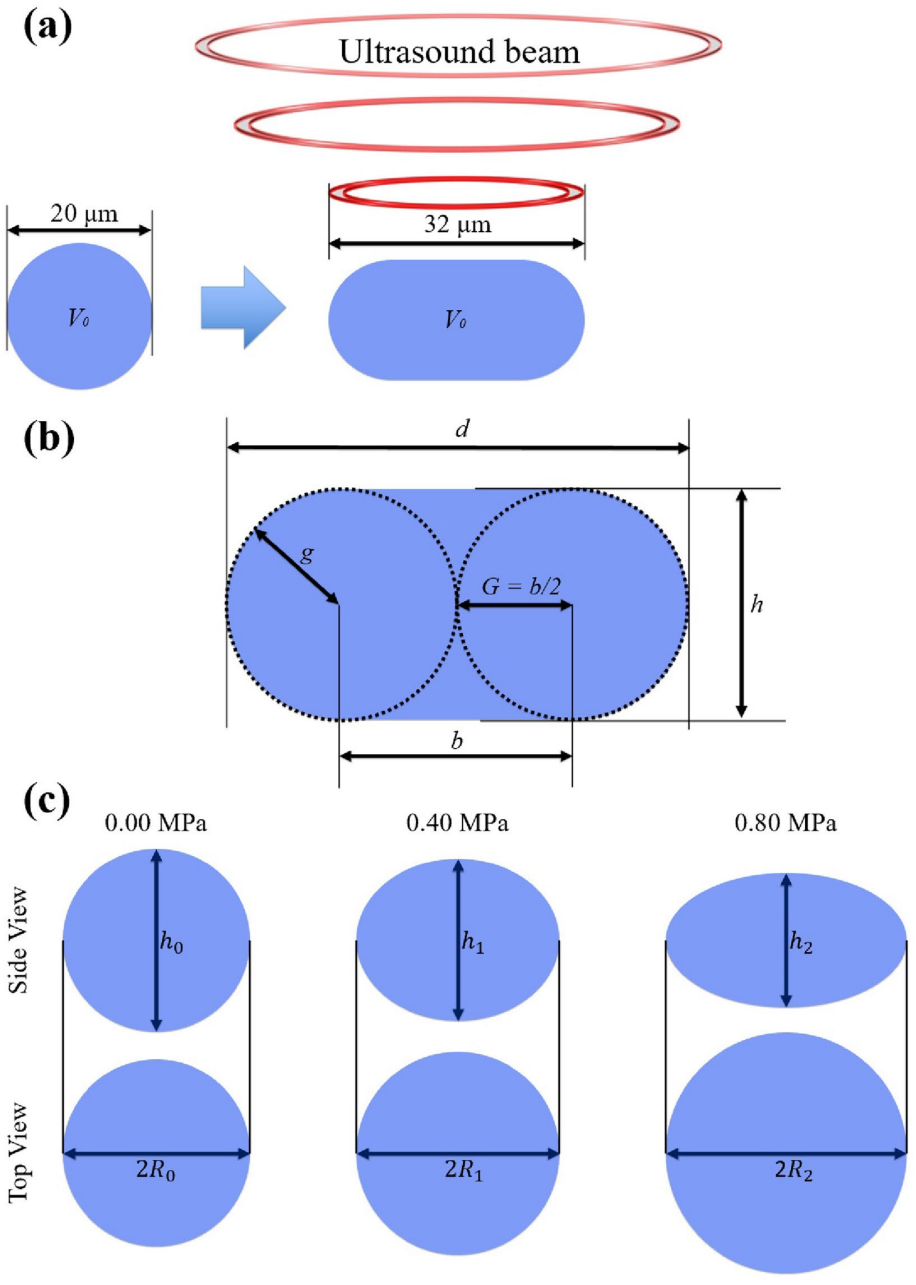
Schematic of the mathematical cell deformation model. (**a**) The initial cell shape is spherical and gradually flattens under SBATs. (**b**) Two circles drawn within the deformed cell represent the height of the cell after deformation $h = 2g$, the radius of the two circles *g*, the diameter of the deformed cell $d = 2G + b$ ($G = b/2$), and the distance of the centers of two circles *b*. (**c**) Young’s modulus *E* is inversely proportional to changes in *h*, which is measured in the vertical direction of the pressure *P* ($E \propto \frac{1}{\Delta h}$). However, the existing method assumes that changes in cell areas ($A \simeq \pi R^2$, where $2R=d$) are directly proportional to pressure, and the average ratios of the changes are inversely proportional to the Young’s modulus ($E \propto \frac{1}{\Delta A}$). Lim et al.^41^ created reference points using $E \propto \frac{1}{\Delta h}$, while they estimated the Young’s modulus using the reference points and $E \propto \frac{1}{\Delta A}$.

Since the two suppositions are not in accordance with the definition of Young’s modulus^44^, they cause uncertainty in the cell deformability estimation. Figure 1c presents the side view and top view of the cell deformation according to pressure levels. An expansion of the cell areas may be correlated to the cell deformation. However, the cells are not perfect spheres or ellipsoidal solids, and in practice, their shapes are nonuniform. Therefore, both $\Delta A = \frac{(A_1 - A_0)/A_0}{P_1 - P_0} = \cdots = \frac{(A_n - A_0)/A_0}{P_n - P_0}$^41^ and $\Delta h = \frac{(h_0 - h_1)/h_1}{P_1 - P_0} = \cdots = \frac{(h_0 - h_n)/h_n}{P_n - P_0}$^45^ cannot be valid, where $A_n$ and $h_n$ indicate cell area and height, respectively, when the *n*-th pressure level $p_n$ is inflicted.

**Figure 2:**
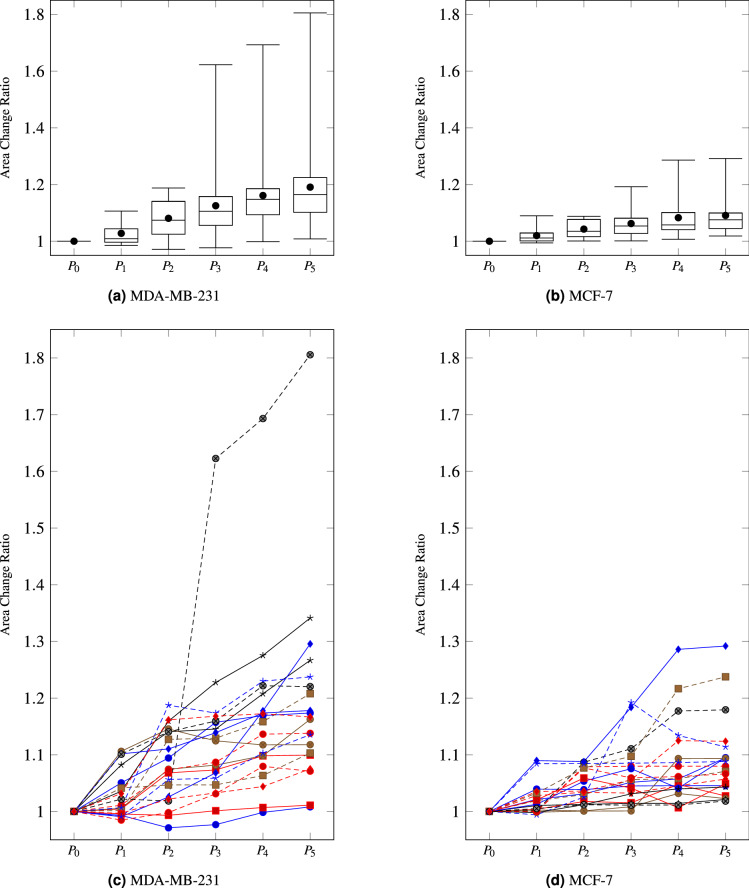
Distributions of area change ratios of cancer cells. (**a**,**b**) present distributions of manually measured area change ratios of the two types of cancer cells (20 cells for each type), MDA-MB-231 and MCF-7, respectively. The three horizontal lines indicate the first, second, and third quartiles of the area change ratios. The tops and bottoms of the whiskers refer to the maxima and minima of area change ratios, respectively. Circular dots denote average area change ratios. (**c**,**d**) show the manually measured area change ratios of each cell, individually. These figures exhibit that cell area changes are not proportional to pressure levels and have diverse tendencies even in a cell-type. In addition, these figures reveal that differences in the area change ratios between the two types are as distinct as we can distinguish types of cancer cells according to the ratios.

For the simplification, let suppose that the cells are in a solid phase with homogeneous and elastic incompressible properties and in the viscoelastic solid model in which the time factor is ignored. However, even if we ignore the permeability of cell membranes and assume that cell volumes are constant, it is difficult to assure that both the cell area changes on the top view ($\Delta A$) and the cell height changes from the side view ($\Delta h$) are directly proportional to the pressure; “Deformation data collection with SBAT” Section presents more details for this point. Thus, the existing method based on the average ratios of the area changes ($\Delta A$) is not reasonable, and we must consider area changes at each pressure level ($\Delta A_n = \frac{(A_n-A_0)/A_0}{P_n - P_0}$) independently to model non-linear correlations of $\Delta A_n$ with $P_n$. As shown in Fig. 2, cell deformation is not significant at low pressure levels, and it becomes accelerated from a certain pressure level and slows down at high pressures.

Most methods for manually measuring Young’s modulus assume that height changes ($(h_0 - h_n)/h_n$) are correlated with deformability (*E*), $E \propto \frac{1}{\Delta h} = \frac{P_n - P_0}{(h_0 - h_n)/h_n}$. However, it is difficult to observe the heights of the cells. Thus, Yokokura et al.^45^ attempted to estimate height changes using diameter changes: $\frac{A_n}{A_0} \propto \frac{R_n^2}{R_0^2} \propto \frac{h_0}{h_n}$, where $R_n$ indicates the cell diameter on $P_n$. For the estimation, they used a physical cast to fix the shapes of the cells. In addition, Lim et al.^41^ assumed $E \propto \frac{1}{\Delta A} = \frac{P_n - P_0}{(A_n-A_0)/A_0}$. They measured $\Delta A$ on multiple pressure levels and averaged them to make the estimated Young’s moduli more accurate. Both methods ignored that cell deformation is not directly proportional to pressure levels. Additionally, because these methods measure $R_n$ or $A_n$ manually, the measuring process is labor-intensive and the subjectivity of operators can be implicated.

This study attempts to solve these problems by employing machine learning techniques. Simply speaking, the problems are (1) unknowingness of correlations between Young’s modulus and cell area changes ($(A_n - A_0)/A_0$) according to changes in pressure ($P_n - P_0$) and (2) absence of methods for (semi-) automatically measuring area changes $(A_n - A_0)/A_0$. We solve these problems as follows:

- *Approximating* $E = f(\Delta A_1, \ldots , \Delta A_n)$ There are correlations between *E* and $\langle \Delta A_1, \ldots , \Delta A_n \rangle$. However because of the nonuniform shapes of cells, it is difficult to infer a function $E = f(\Delta A_1, \ldots , \Delta A_n)$ from the physical characteristics of cells and the definition of *E*. Thus, we first reduce the parameters of this function by taking photomicrographs of cells at fixed pressures. Then, we approximate $E = f(\frac{A_1 - A_0}{A_0}, \ldots , \frac{A_n - A_0}{A_0})$ using multilayer perceptron (MLP), which is effective for emulating complicated functions.
- *Measuring *$\Delta A$ The existing study^41^ found cell boundaries manually and counted the number of pixels within the cell boundaries. We automate this process to reduce its labor intensiveness and exclude the subjectivity of operators. We train a convolutional neural network (CNN) model to measure the normalized cell deformation ($\frac{A_n - A_0}{A_0}$) by comparing cell boundaries after inflicting a certain degree of pressure ($P_n$) with the boundaries before inflicting.

**Figure 3:**
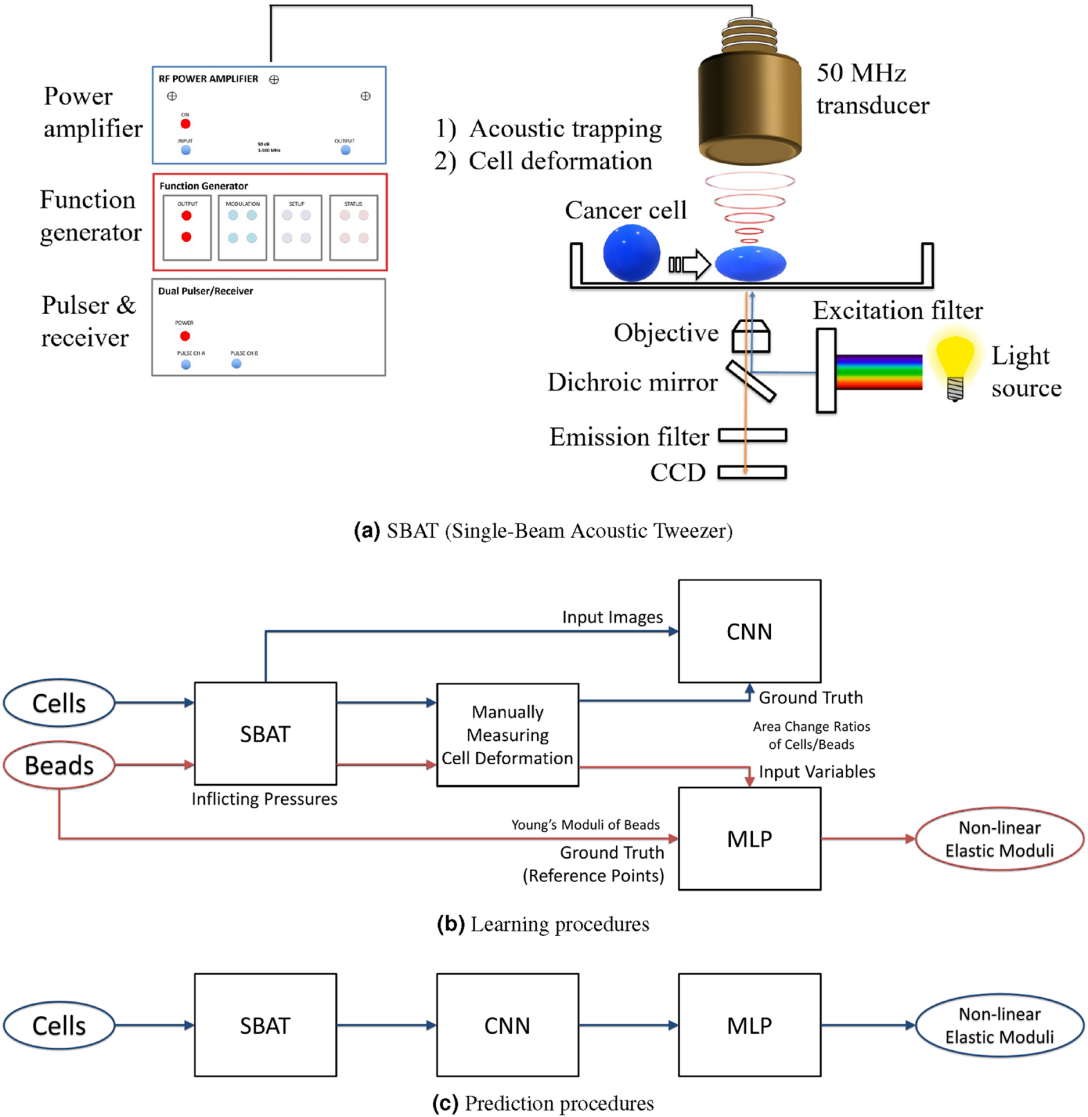
Overview of the proposed system for the automated cell deformability estimation using SBAT, CNN, and MLP. (**a**) SBAT traps and deforms the cancer cell to measure its elasticity and provides photomicrographs for the learning and prediction processes of CNN and MLP. (**b**,**c**) CNN measures cell area changes by analyzing the cell photomicrographs, and MLP estimates the non-linear elastic modulus of cells by learning correlations of the area changes with pressure levels. (**b**) The SBAT is applied to cells and beads to cause deformation. We trained the CNN and MLP models by quantifying the deformation manually. We used Young’s moduli of the beads as reference points for quantizing cells’ elasticity. (**c**) On prediction, the SBAT, CNN, and MLP compose a pipeline for (semi-) automatically estimating non-linear elastic moduli of the cells.

Figure 3 presents a conceptual overview of the proposed system, which is a combination of SBAT, CNN, and MLP. Cell deformability estimation is conducted by executing the three components sequentially, as shown in Fig. 3c. However, learning procedures of the neural networks are slightly complicated, because we employ the standardized reference data of Young’s moduli of beads instead of directly obtaining the accurate Young’s moduli of cells. Thus, the MLP is trained using beads for which we know the exact Young’s moduli, while we train the CNN using photomicrographs of real cancer cells and their area changes measured manually on multiple pressure levels, as described in Fig. 3b.

Additionally, this study supposes cell deformation and pressure have non-linear correlations, as observed in live cells (Fig. 2). However, Young’s modulus is defined based on the assumption that the deformation is linearly proportional to pressure. Therefore, although the proposed system quantifies elasticity of live cells by learning correlations of Young’s moduli of beads with their deformation according to pressure, it had better not to name as Young’s moduli due to the issue of linearity. Thus, we use the term of ‘non-linear elastic modulus’ for describing the elasticity degree of cells in this study.

The absence of the ground truth for real cells causes problems in evaluating the proposed system because it is difficult to assess the accuracy of non-linear elastic moduli estimated by the system. Thus, we first validated the effectiveness of the CNN and MLP models individually. The CNN model was evaluated based on two types of cancer cells, and five types of beads were used to evaluate the MLP model. We evaluated the practicality of the entire system by validating whether non-linear elastic moduli estimated by the proposed system can distinguish invasive cancer cells from noninvasive ones.

Lim et al.^46^ successfully classified cancer cells based on their deformability using CNN; however, it did not achieve the absolute value of elastic modulus because of the lack of calibrated elastic modulus data. We extended the application of SBAT and machine learning technology to estimate the elastic modulus of cancer cells, which directly correlates to their metastatic potential and provides a guide during cancer treatment. To the best of the author’s knowledge, this is the first time automated methods have been developed for measuring cell elasticity using ultrasound devices and machine learning.

The remainder of this paper is organized as follows. In “Automated cell deformability estimation” Section, we present our data collection procedures and propose methods for measuring cell area changes and estimating non-linear elastic modulus. In Section Evaluation, we validate the accuracy and practicality of the proposed system.  “Conclusion” Section presents concluding remarks and future research directions.

## Automated cell deformability estimation

This section presents a system for automatically measuring the non-linear elastic modulus of live cells (i.e., a quantized value of cell deformability). The system consists of three parts: (1) collecting cell deformation data (SBAT), (2) measuring the degree of cell deformation (CNN), and (3) estimating non-linear elastic moduli of cells (MLP), as shown in Fig. 3. Using the SBAT, we deform the cells by inflicting specific pressures. After capturing photomicrographs of the deformed cells, we preprocess the cell images to reduce noise and emphasize the cell deformation. The CNN is used to quantify degrees of deformation. Finally, we apply the MLP to estimate non-linear elastic modulus by analyzing the deformation degrees on multiple pressure levels.

### Deformation data collection with SBAT

This section explains how we captured the photomicrographs of cells and beads using the SBAT, how we measured the area change ratios of cells and beads on the photomicrographs, and how we measured the real Young’s moduli of the beads.

#### SBAT

**Figure 4:**
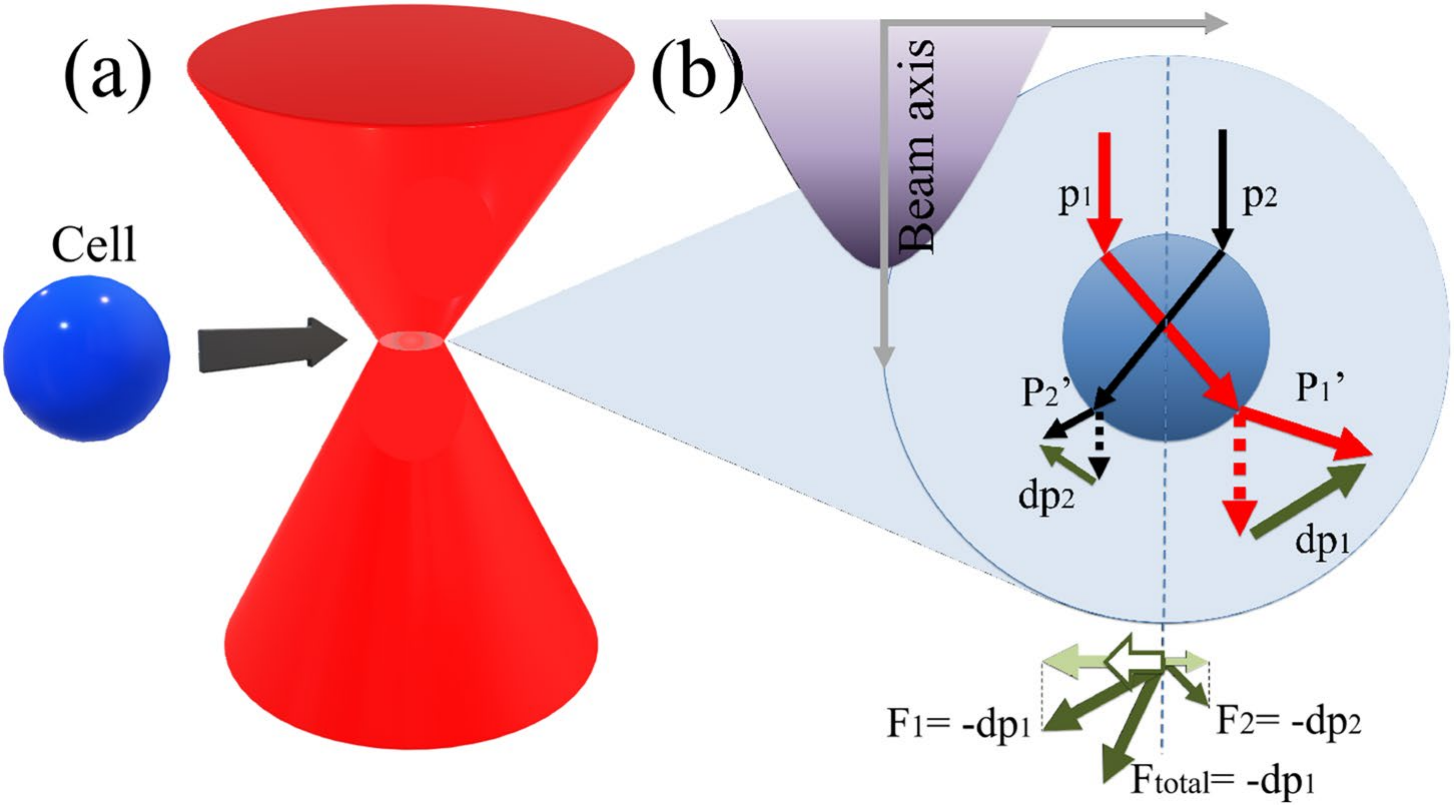
Mechanism of SBAT. The momentum of two rays (red and black arrows) with different intensities propagating through the cell. Ftotal represents the net force, and the white arrow indicates the direction of the cell movement along the lateral direction in this experiment.

The physical mechanism of SBAT is similar to that of the optical tweezers, as shown in Fig. 4. When a Gaussian ultrasound beam is incident on a single cell, the rays refract into the cell. Rays ($p_1$) nearest to the beam axis are more intense than the rays ($p_2$) on the beam edge. The direction of the ultrasound rays is changed at each refraction, which results in the conservation of momentum while imparting a gradient force on the cell. The force generated from the directional change of the ray’s momentum has components in the forward and side directions. $F_1$ and $F_2$ represent the forces imparted to the cell by $p_1$ and $p_2$, respectively. Ftotal is the sum of these two vectors, and it is directed toward the beam axis. Because this experimental setup in the presence of a petri dish considers only a transversely trap, the cell is displaced to the ultrasound focus along the lateral direction (white arrow in Fig. 4). If the cell is located at the ultrasound focal point, there is no net gradient force presented because the ultrasound refracts symmetrically.

#### Transducer fabrication

**Figure 5:**
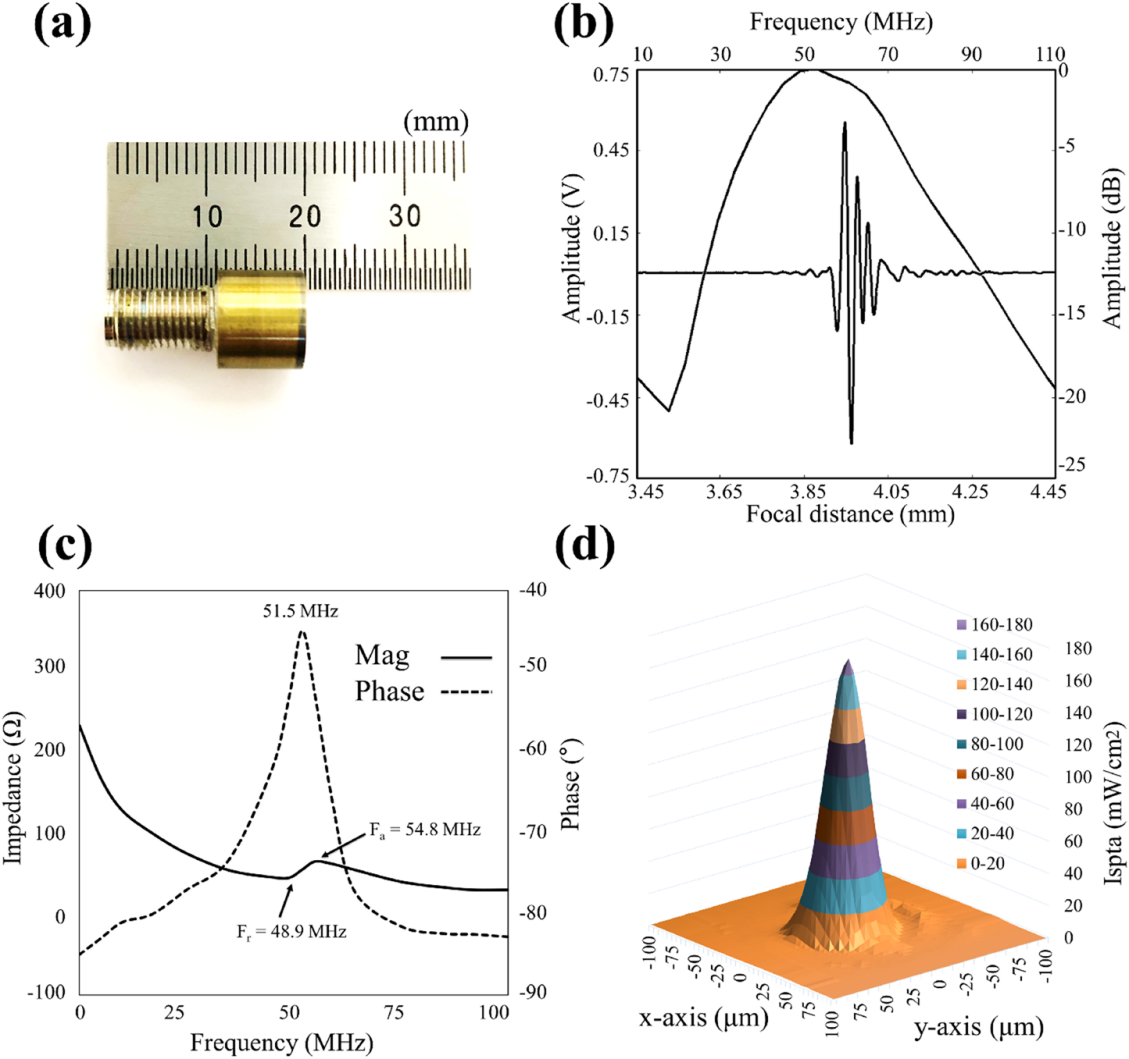
Profile of the home-made ultrasonic transducer (**a**) Photograph of the transducer; (**b**) Pulse-echo response and frequency spectrum; (**c**) The electrical impedance of the transducer (the solid line (the impedance magnitude) and the dotted line (phase angle)); and (**d**) Acoustic intensity field of the ultrasonic transducers. MATLAB R2020a (https://www.mathworks.com/products/new_products/release2020a.html) was used to create (**b**,**c**).

A customized high-focused 50 MHz transducer was developed and fabricated in our laboratory, as shown in Fig. 5a^46^. The fabricated transducer comprised three layers: a 61-μm-thick piezoelectrical layer (lithium niobate), 9-μm-thick matching layer (2–3 μm silver power epoxy), and 1-mm-thick backing layer (E-solder silver epoxy). The acoustic stack was turned down to 5 mm using a lathe and press-focused at 4 mm using a bearing ball. Further details on the transducer design and fabrication procedure are provided in Lim et al.^46^.

A commercial pulser-receiver JSR (DPR 500, Pittsford, NY, USA) was used to verify the performance of the custom-fabricated 50 MHz transducer. The JSR was connected to the transducer and exited the electrical impulses at a 500 Hz repetition rate at 50 dampings; a quartz reflector was used in this test. Figure 5b shows the results of the pulse-echo response and frequency spectrum of the transducer. The center frequency was 51 MHz, and the $-6~dB$ fractional bandwidth was 80%. We measured the electrical impedance of the transducer using an Agilent 4991A impedance analyzer (Agilent Technologies, Santa Clara, CA). Figure 5c shows the frequency dependence of the impedance and the phase angle. The resonance and anti-resonance frequencies were found to be 48.9 and 54.8 MHz, respectively. The magnitude and phase angle of the electrical impedance of the transducer were 55 $\Omega$ and $-\,45^{\circ }$, respectively. The acoustic intensity field of the ultrasonic transducer was measured using a needle-type hydrophone (HPM04/01, Precision Acoustics, United Kingdom). The − 3 dB lateral beam width was 32 μm. The acoustic intensity field of the ultrasonic transducer was measured using a needle-type hydrophone (HPM04/01, Precision Acoustics, United Kingdom) as shown in Fig. 5d. The driving conditions were as follows: frequency of 50 MHz, input peak to peak voltage of 25 $V_{pp}$, cycle number of 10, and pulse repetition frequency (PRF) of 1 kHz. The − 3 dB lateral beam width was measured to be 32 μm.

#### Cell preparation

MDA-MB-231 and MCF-7 cell lines were purchased from ATCC (Manassas, VA, USA) and maintained in complete growth medium (RPMI, 10% fetal bovine serum, 10 mM HEPES, 2 mM L-glutamine, 1 mM sodium-pyruvate, 0.05 mM 2-mercaptoethanol, and 11 mM D-glucose). Both cells were seeded at a density of 1.3 $\times$ 105 $\mathrm{cells}/\mathrm{cm}^2$ and cultured in 5% $\hbox {CO}_2$ at 37 $^{\circ }\mathrm{C}$. Phosphate buffer solution (PBS) was purchased from Invitrogen (Grand Island, NY) for cell washing before dissociation. Trypsin-ethylenediaminetetraacetic acid (trypsin-EDTA) solution was purchased from Invitrogen (Grand Island, NY) to detach cells from the Petri dish. Trypsin-EDTA was incubated with cells at 37 $^{\circ }\mathrm{C}$ for less than 5 min. 2 mL of media was poured into a Petri dish to maintain cell suspension. The cell viability assays after SBAT confirmed that the cells remained viable.

#### Cell deformation using SBAT

The transducer was fixed and controlled by a three-axis motorized stage (SGSP20, Sigma KOKI Co., Japan). A cell was located at the focal point of the ultrasound beam. A pulser-receiver (5910PR; Olympus, Center Valley PA, USA) was used for the alignment. To deform the cell, a 50 MHz sinusoidal burst signal generated by a function generator (Stanford Research Systems, Sunnyvale, CA, USA) and amplified by a 50 dB power amplifier (525LA, ENI, Rochester, USA) was driven on the transducer. The duty cycle and pulse repetition frequency were set to 500 cycles and 1 kHz, respectively. The input peak-to-peak voltages were set to 0.00, 4.74, 9.48, 14.22, 18.96, or 23.70 V. The cell deformations under the SBAT were observed using an inverted microscope (Olympus IX-71, Center Valley, PA, USA), and the photomicrographs of deformed cells were captured using a CMOS camera (ORCA-Flash2.8, Hamamatsu, Japan), as shown in Fig. 3a. Recording frame rate was 15 *fps*. The images were captured and analyzed when applied acoustic pressure was 0.00, 0.23, 0.43, 0.63, 0.82, and 1.00 MPa. When acoustic pressure was increased, the cell was deformed right away. Total time of cell deformation from 0.00 to 1.00 MPa was 60 s.

#### Manual measurement of cell deformability

After deformation experiments, the area of the cells was measured using ImageJ (NIH, Bethesda, MA, USA). Edges that define a cell boundary were clicked, and a polygon shape around a cell was formed, connecting the line. The polygon area and cell surface area were calculated. The cell area increased as the acoustic pressure increased. For each sample, the captured images were analyzed at input voltages of 0.00, 4.74, 9.48, 14.22, 18.96, and 23.70 $V_{pp}$ (corresponding acoustic pressures: 0.00, 0.23, 0.43, 0.63, 0.82, and 1.00 MPa).

#### Measurement of Young’s modulus of beads

*E* was determined from the shape of the deformed cell. In particular, the diameter or the area of the deformed cell following compression can be obtained from the experiment and is shown to be inversely proportional to *E*, as proven mathematically below. While deforming the cell with an ultrasound beam (pressing a cell with radiation forces), the cell was flattened as shown in Fig. 1a,b. The volume of the cell ($V_0$) can be expressed as:

1 $$\begin{aligned} V_0&= \frac{4}{3} \pi g^3 + \pi Gg (2G + \pi g), \end{aligned}$$

2 $$\begin{aligned} d&= \left( 1 - \frac{\pi }{4} \right) h + \sqrt{\frac{\pi ^2}{16} h^2 + \left( \frac{4V_0}{\pi h} - \frac{2}{3} h^2 \right) } = \sqrt{\frac{4A}{\pi }}, \end{aligned}$$

where *h*, *g*, *d*, and *b* denote the height of the cell after deformation, radius of two circles of the deformed cell, diameter of the deformed cell, and distance between the centers of the two circles, respectively. *G* equals *b*/2 after deformation, and *d* was equal to *h* (where $G= 0$) at initial condition (before deformation). Two circles drawn within the deformed cell represent the height of the cell after deformation ($h = 2g$) and the diameter of the deformed cell ($d = 2G+b$). As shown in Eq. (2), diameter of the deformed cell (*d*) is proportional to the area change (*A*) in the top view. Thus, it proved that the diameter or the area of the deformed cell following compression is shown to be inversely proportional to *E*, and *E* is estimated by the cell area expansion as:

3 $$\begin{aligned} E = \frac{P}{\varepsilon _A} = -\frac{P}{(A_0 - A)/ A_0} \end{aligned}$$

where $A_0$ is the original area of the cell, and *P* denotes the applied pressure. Thus, the Young’s modulus is assumed to be estimated by considering the change in the deformation area.

**Figure 6:**
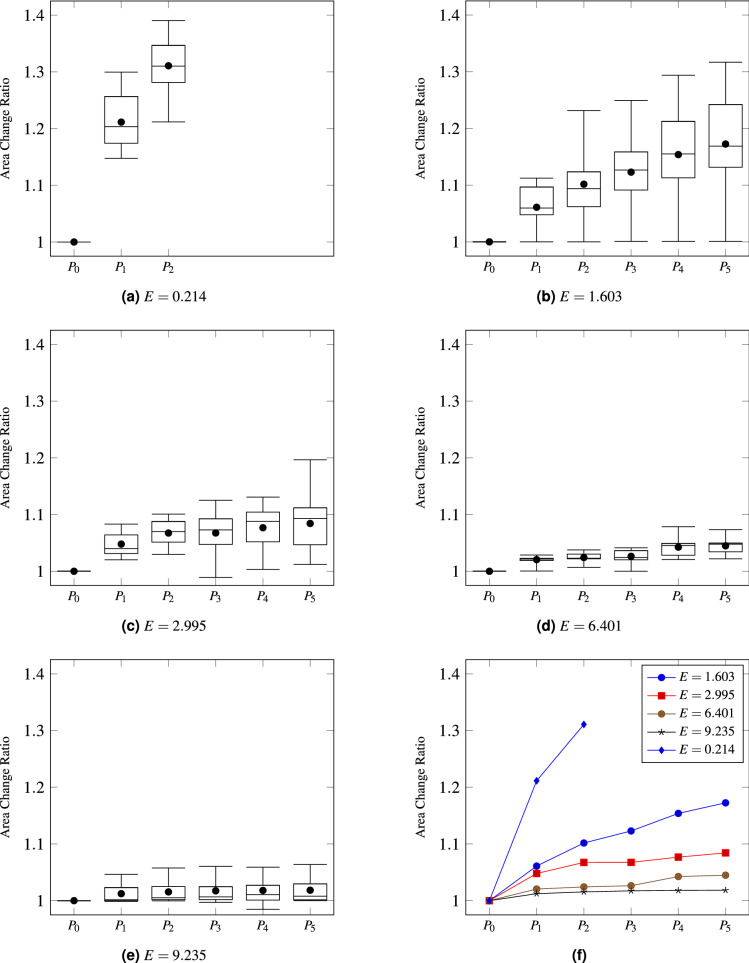
Distributions of area change ratios of beads according to their Young’s moduli. (**a**–**e**) present manually measured area change ratios of the five types of beads (20 beads for each type), $E = 0.214$, 1.603, 2.995, 6.401, and 9.235, respectively, in the same manner with Fig. 2 (**a**,**b**). (**f**) shows a comparison between the average area change ratios of each bead. The six figures exhibit that area changes of beads are not linearly proportional to pressure levels, as with live cells.

In our experiment, the deformation rate of the cell under the SBAT pressure was measured. Recently, agarose based cell or tissue mimicking phantom has been widely used in the biomaterial field^47,48^. Thus, in our study, agarose hydrogel spheres (AHBs) were served to standardize the biomechanical characteristics of the cells and provided the reference data between the deformability and *E*. Since the mechanical properties of agarose hydrogel spheres depend on their agarose concentration, agarose hydrogel spheres in 0.1, 0.3, 0.6, 0.9, and 1.2% agarose concentrations were purchased from Particle-works (Royston, United Kingdom). The average diameter of both cells and spheres size is about 15 μm. The *E* of AHBs with five different agarose concentrations was measured by a micropipette aspiration technique (MAT), which is the method for investigating the mechanical properties of cells and cell-sized microspheres^49^. A glass capillary containing a filament (GD-1, Narishige, NY), a vertical micropipette puller (PC-10, Narishige, NY), and a pressure controller (ez-gSEAL 100B, Neo biosystem, CA) were used for this measurement. Detailed procedure is described in our previous study^41^. After the MAT, we could confirm that Young’s modulus was directly proportional to the amount of agarose in the sphere. Those beads underwent a deformation test with SBAT similar to the cell deformation test. Based on the analytical comparison of deformability levels between the cells and the AHBs, the *E* of cells could be indirectly measured by interpolating the Young’s moduli of the AHBs. Further details on the quantification of *E* of cancer cells can be found in Lim et al.^41^. Figure 6 presents area change ratios of the beads according to pressure levels and their *E*. The area change ratios and pressure levels were not linearly proportional. Also, the beads’ area changes had high variance as with cells (Fig. 2). Therefore, these cell-mimicking beads, which are manufactured to have the specified Young’s modulus, can provide references for quantifying the elasticity of live cells despite the non-linearity and high variance of cell area change ratios.

### Preprocessing cell photomicrographs

**Figure 7:**
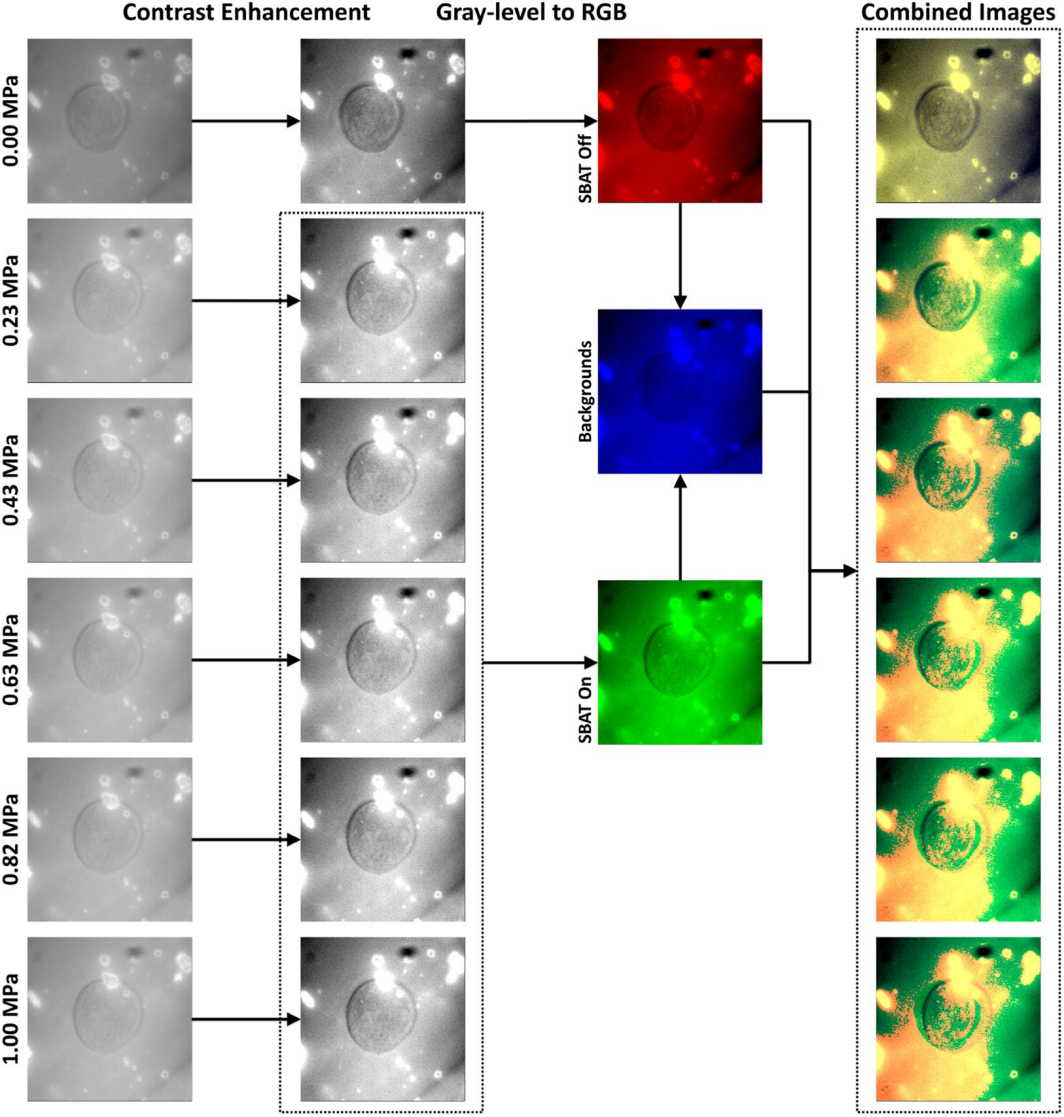
Proposed preprocessing procedures for emphasizing changes in cell boundaries. The first column shows cell photomicrographs captured at six pressure levels: 0.00, 0.23, 0.43, 0.63, 0.82, and 1.00 MPa. We enhance the contrast of the photomicrographs, as shown in the second column. Subsequently, we attempt to represent the deformation degrees with a single three-channel image. The photomicrographs at 0.00 MPa are placed in red channels, and the remaining images are used as green channels. The blue channels mark the background areas by averaging the photomicrographs at all pressure levels. The last column shows the preprocessing results. We preprocessed the photomicrographs and drew this figure by using OpenCV 4.2.0 (https://opencv.org/opencv-4-2-0/) with Python 3.7 (https://www.python.org/downloads/release/python-370/).

Our photomicrographs are considerably noisy because the SBAT system does not allow us to use condensers located above the stage and below the light source in an inverted microscope. The ultrasonic transducer should be located above the samples, which is the intrinsic limitation of our SBAT system regarding its unclear images. If operators are not used to adjust the focal distances, the cell boundaries will not be clear. Noisy and blurry images are not helpful for comparing the inner areas of the boundaries. Thus, we propose a preprocessing method for emphasizing the changes between $P = 0.00~\mathrm{MPa}$ and $> 0.00~\mathrm{MPa}$ cases, as shown in Fig. 7.

The photomicrographs are gray-level images, which are single-channel. We compose three-channel images that consist of (1) cell areas before the deformation, (2) after the deformation, and (3) background areas. The combined images enable the CNN models to compare the three areas using one input image. First, we enhance the contrast of the photomicrographs by normalizing pixel values to [0, 255]. The second row of Fig. 7 shows results of the contrast enhancement. Second, we find background areas by comparing pixels on SBAT off images with pixels on SBAT on images. We assume that background areas are more consistent than cell areas, whether the SBAT is on or off. The three channels reveal changes in cell boundaries as:

4 $$\begin{aligned} D= \begin{bmatrix} 70&{}70&{}\mathbf{90} &{}50&{}50\\ 70&{}60&{}60&{}\mathbf{90} &{}50\\ 60&{}70&{}60&{}\mathbf{90} &{}40\\ 60&{}70&{}\mathbf{90} &{}50&{}70\\ 70&{}60&{}\mathbf{90} &{}50&{}\mathbf{90} \end{bmatrix}, ~~ N= \begin{bmatrix} 70&{}\mathbf{90} &{}50&{}50&{}40\\ 60&{}60&{}\mathbf{90} &{}50&{}40\\ 70&{}70&{}\mathbf{90} &{}40&{}40\\ 60&{}\mathbf{90} &{}50&{}30&{}80\\ \mathbf{90} &{}50&{}40&{}40&{}\mathbf{90} \end{bmatrix}, ~~ B= \begin{bmatrix} 70&{}\mathbf{80} &{}\mathbf{70} &{}50&{}{45}\\ {65}&{}60&{}\mathbf{75} &{}\mathbf{70} &{}{45}\\ {65}&{}70&{}\mathbf{75} &{}\mathbf{65} &{}40\\ 60&{}\mathbf{80} &{}\mathbf{70} &{}{40}&{}{75}\\ \mathbf{80} &{}{55}&{}\mathbf{65} &{}{45}&{}90 \end{bmatrix}, \end{aligned}$$

where *N* and *D* indicate parts of the photomicrographs captured before and after the deformation, respectively, and *B* denotes the background channel, which is an average of *N* and *D*. When the left sides of the partial images are cell areas, we can find cell boundaries on *N* and *D* that have higher pixel values than the others. If we compare *D* with only *N*, it is difficult to determine whether the bottom-left corner of *N* is noise. Using *B*, we can determine areas that have significantly changed after inflicting pressures.

In contrast to other research domains, culturing cells consume many resources and human efforts. Thus, it is difficult to compose a large-scale dataset. This study is conducted on 40 cells and 6 photomicrographs for each cell. Using data augmentation, we attempt to avoid the over-fitting issue. Furthermore, the photomicrographs can be noisy and out of focus. The data augmentation can make our model robust to these problems. With ImageDataGenerator in Keras, we generate augmented cell images by rotating, flipping, zooming, rescaling, and translating the original images.

### Cell area change estimation

To measure area changes, the most convenient and popular approach may be segmenting photomicrographs based on cell boundaries and counting the number of pixels inside the boundaries. Nevertheless, owing to the noise in photomicrographs and the diversity of cells, it is difficult to develop a segmentation method for general cells. Black box models, such as CNN, can be effective for this type of problem. When we have novel cells, CNN can learn convolutional filters that are adequate for the cells.

For a cell, we capture the photomicrographs on six pressure levels. We denote the levels as $P_0$ to $P_5$. Thus, on the *n*-th pressure level ($P_n$), we attempt to emulate the ratio of the cell area on $P_n$ for on $P_0$ ($\Delta A(c_i, P_n)$). When $A(c_i, P_n)$ indicates the size of a cell $c_i$ on $P_n$, our CNN model for measuring area changes can be described as:

5 $$\begin{aligned} \frac{A(c_i, P_n)-A(c_i, P_0)}{A(c_i, P_0)} = \Delta A(c_i, P_n) \simeq f(X_{i,N},X_{i,0};\theta _A) \end{aligned}$$

where $X_{i,N}$ indicates a photomicrograph of $c_i$ taken on $P_n$, $f(\cdot ,\cdot ;\theta _A)$ denotes the CNN model, and $\theta _A$ is a parameter set of the model.

**Figure 8:**
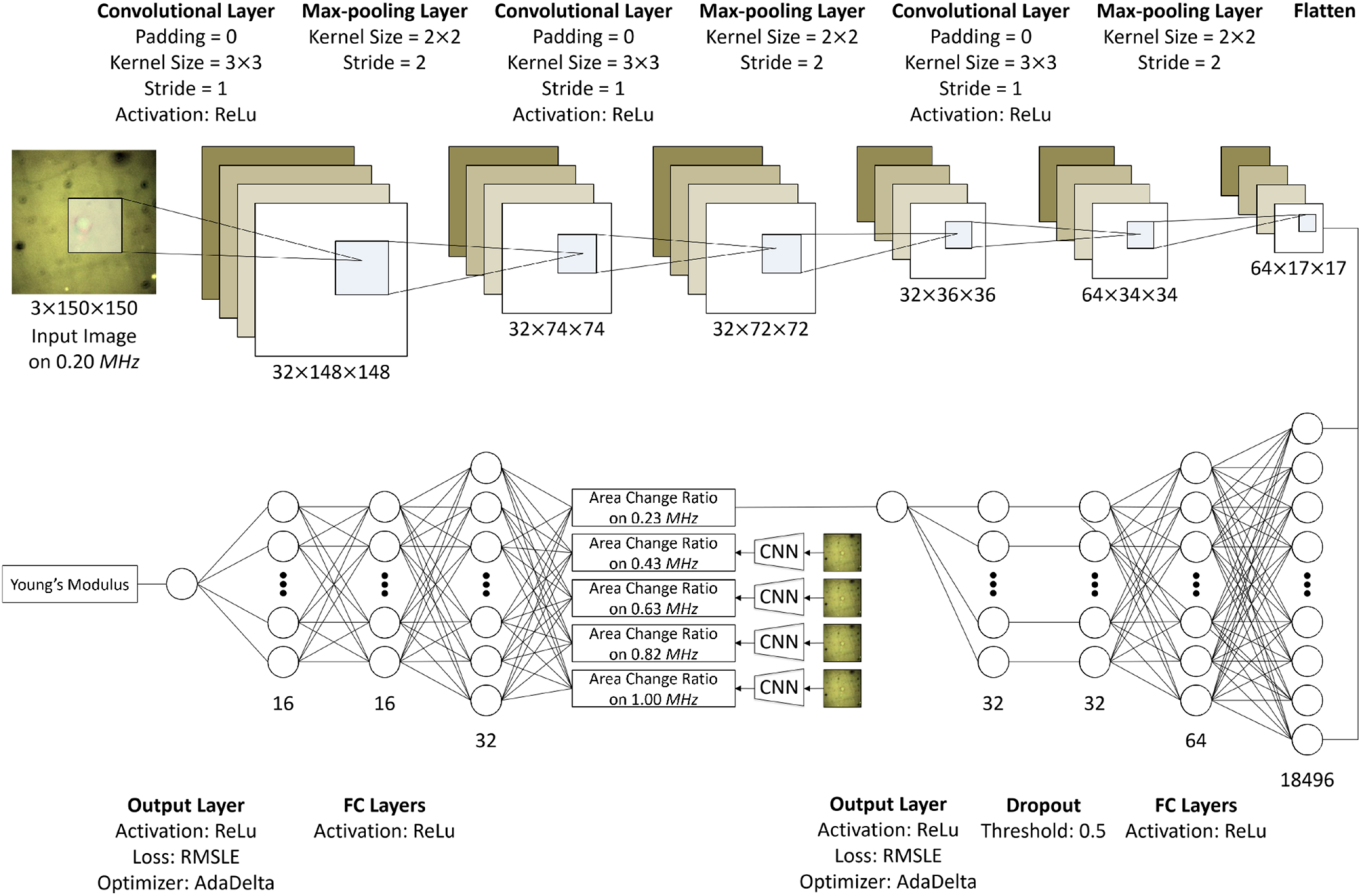
Proposed models for measuring area change ratios and estimating non-linear elastic moduli. The CNN model for area changes consists of three 2-D convolutional layers and two fully connected layers. The MLP model for non-linear elastic moduli consists of two fully connected layers.

Figure 8 describes a structure of the proposed CNN model that consists of three convolutional layers, three max-pooling layers, three fully-connected layers, and one dropout layer. The activation functions of the convolutional layers and fully connected layers are rectified linear unit (ReLu), and the threshold of the dropout layer is set as 0.50. As a loss function, we applied mean absolute error (MAE), mean squared error (MSE), root mean squared error (RMSE), mean absolute percentage error (MAPE), mean square logarithmic error (MSLE), and root mean squared logarithmic error (RMSLE).

In the evaluation in “Accuracy of measuring cell area changes” Section, MAPE, MSLE, and RMSLE achieved a considerably higher accuracy than the others, and RMSLE was the best among them. Because the area change ratios of cells have small values $\in [1,2]$ as shown in Fig. 2, the range of values may make the ratio-based loss functions outperform deviation-based ones.

The parameter set ($\theta _A$) is updated according to the directions and sizes of the gradients of the model’s objective function to minimize the loss. The objective function can be formulated as:

6 $$\begin{aligned} \mathcal {L}_A (\theta _A) = \left[ \frac{1}{I \times N} \sum _{\forall c_i, \forall P_n}{ (\log ( \Delta A(c_i, P_n) + 1) - \log (f(X_{i,n},X_{i,0};\theta _A) + 1))^2} \right] ^{\frac{1}{2}}, \end{aligned}$$

where *I* indicates the number of cells and *N* refers to the number of pressure levels. To update $\theta _A$ using the gradients, various methods (optimizers) have been proposed. We applied the stochastic gradient descent (SGD)^50^, RMSProp (http://www.cs.toronto.edu/~tijmen/csc321/slides/lecture_slides_lec6.pdf), AdaGrad^51^, AdaDelta^52^, and Adam^53^ on our CNN model, and AdaDelta achieved the best accuracy. Parameter updating with AdaDelta can be formulated as:

7 $$\begin{aligned}&G := \gamma G + (1-\gamma ) (\nabla _{\theta _A} L_A(\theta _A))^2, \nonumber \\&\Delta _{\theta _A} = \frac{\sqrt{s + \varepsilon }}{\sqrt{G + \varepsilon }} \nabla _{\theta _A} L_A(\theta _A), \nonumber \\&\theta _A^* := \theta _A - \Delta _{\theta _A}, ~~ s := \gamma s + (1-\gamma ) \Delta _{\theta _A}^2, \end{aligned}$$

where $\theta _A^*$ denote the updated parameters, *G* refers to the average of the squared gradients, *s* denotes the average of the squared parameters, $\gamma$ indicates a momentum factor for the gradients, $\varepsilon$ is used to prevent division by zeroes, and $:=$ denotes the assignment operator.

Additionally, although various CNN-based cell segmentation models, such as U-Net^54^, have been proposed, these models require training as a large number of parameters as their performance. However, since this study deals with live cells, it is challenging to compose a sizeable live-cell dataset satisfying both our experimental setting and scale for training the large parameter set. Therefore, in this study, we designed a relatively shallow CNN regression model for directly estimating area change ratios of cells. When the scale of a dataset is restricted, we expect that the proposed shallow CNN can exhibit reasonable accuracy. At the same time, the state-of-the-art deep CNN models are difficult to be properly trained.

Also, various conventional cell segmentation tools are used in biology studies, such as ImageJ plugins for fluorescence images. However, in our case, we used the cell photomicrographs taken under bright field, not fluorescent cell imaging, so intensities of the background image and the cell image were very similar, causing unclear cell boundaries. Another issue is that our SBAT system has the intrinsic limitation that it is not able to use the condenser, which illuminates the cell. This is because the ultrasonic transducers should be located above the stage, as shown in Fig. 3. As a result, the amount of concentrated light source that illuminates the specimen is relatively less than that of the general inverted microscope, and this also causes unclear cell boundaries. It was difficult to recognize the morphological gradient, which shows the boundaries of cells, using morphological segmentation plugins in ImageJ that underestimated the cell area. Therefore, we used the proposed CNN model rather than the conventional image processing methods for cell photomicrographs.

### Non-linear elastic modulus approximation

Young’s modulus is a standard assessment criterion of deformability. However, we cannot determine the exact correlations between *E* and $\Delta A (c_i)$. Although Lim et al.^41^ supposed that $\Delta A(c_i, P_1) = \cdots = \Delta A(c_i, P_n)$ and $\Delta A(c_i, P_n) \propto 1/E$, the area change ratios of cells are not directly proportional to pressure, as shown in Fig. 2. Thus, this study does not attempt to reveal the correlations, and we emulate a function $E(c_i) = f(\Delta A(c_i))$ using the MLP, which is a black box model. Since the approximated $E(c_i)$ is not an average gradient of cell area change ratios for pressures, it is difficult to say that $E(c_i)$ is Young’s modulus of a live cell $c_i$. However, the MLP is trained based on the manually measured Young’s moduli of beads, and we can compare relative deformability of cells with other cells or objects by using the approximation of MLP. Therefore, we call the approximated $E(c_i)$ ‘non-linear elastic modulus,’ as discussed in  “Introduction” Section. Excluding the case $P = 0.00~\mathrm{MPa}$, the area changes on the pressure levels compose a vector given as:

8 $$\begin{aligned} \Delta A (c_i) = \left\langle \Delta A(c_i, P_1), \Delta A(c_i, P_2), \Delta A(c_i, P_3), \Delta A(c_i, P_4), \Delta A(c_i, P_5) \right\rangle . \end{aligned}$$

As shown in Figs. 2 and 6, both cells’ and beads’ area change ratios have non-linear correlations with degrees of inflicted pressures. Also, the area change ratios have high variance even in a bead-type and cell-type. However, the conventional regression models, which find an optimal mathematical expression describing correlation of inputs with outputs, are difficult to model that beads with the same Young’s moduli can exhibit varied area change ratios for the same pressure level. Therefore, we employ MLP to deal with the non-linearity and high variance, although $f(\Delta A(c_i))$ does not have numerous parameters.

Our MLP model can be formulated as $E(c_i) \simeq f(\Delta A(c_i);\theta _E)$, where $\theta _E$ indicates a parameter set of the MLP. The bottom left side of Fig. 8 describes the structures of our MLP model. We use three fully connected layers that consist of 32, 16, and 16 nodes, respectively. To train the MLP model, we applied MAE, MSE, RMSE, MAPE, MSLE, and RMSLE as loss functions and SGD, RMSprop, Adagrad, AdaDelta, and Adam as optimizers. As with the CNN, RMSLE and AdaDelta showed the best accuracy. An objective function of the MLP can be formulated as:

9 $$\begin{aligned} \mathscr {L}_E (\theta _E) = \left[ \frac{1}{I} \sum _{\forall c_i}{ (\log ( E(c_i) + 1) - \log (f(\Delta A (c_i);\theta _E) + 1))^2} \right] ^{\frac{1}{2}}. \end{aligned}$$

The MLP model is trained using area change ratios and the manually measured Young’s moduli of beads and not cells because a mechanical contact is required during the Young’s moduli test of live cells, which leads to damage to the cell membrane. With a damaged cell membrane, the measurement of cell deformability is not reliable. Therefore, as discussed in “Deformation data collection with SBAT” Section, we measured the area change ratios and Young’s moduli of five types of beads on five pressure levels. However, beads with the same Young’s modulus do not exhibit consistent area changes, as shown in Fig. 6. This inconsistency is far more severe in the cells (Fig. 2). This problem comes from both operators and the diversity of cells. Although SBAT is a reliable tool, it is manually operated by humans who can make mistakes; thus, this study attempts to minimize human intervention. Further, both cells and cell-mimicking beads are incompressible micron-sized objects, which result in single-cell variability (individual cells slightly differ in shape and size). Moreover, even though the proposed CNN model has high accuracy, it cannot be more accurate than manually counting pixels.

The proposed MLP model should be able to handle the inconsistency and uncertainty that arises from both the SBAT and CNN models. Thus, we collected area changes of 20 beads for each type and applied 5-fold cross-validation to the 100 samples. To improve the generalization of the model, we employed noises and parameter initialization. Weights on every layer were initialized as random variables with a Gaussian distribution, and biases were initially set as 0. In addition, we injected Gaussian noises into the first two layers.

Furthermore, beads and cells exploded when we inflicted high pressures compared to their deformability, as shown in Fig. 6a. To express the explosion, we examined three approaches: huge values (e.g., 999), zeroes, and negative values. Among these three, negative values yielded the highest accuracy.

## Evaluation

We collected data from 100 beads and 40 cancer cells. We captured (i) photomicrographs of beads and cells by inflicting certain pressures: 0.00, 0.23, 0.43, 0.63, 0.82, and 1.00 MPa. For the beads, we measured (ii) their Young’s modulus manually. Further, we calculated (iii) area changes of the beads and cells on each pressure level manually. Finally, the cancer cells consisted of (iv) two types: MDA-MB-231 (invasive) and MCF-7 (noninvasive).

Using (i) and (iii), we evaluate the proposed CNN model for measuring area changes automatically (“Accuracy of measuring cell area changes” Section). Based on (ii) and (iii), we validate whether our MLP model can emulate $E(c_i) = f(\Delta A(c_i))$ (“Accuracy of estimating non-linear elastic modulus” Section). Finally, (i) and (iv) are applied to examine the practicality of the proposed methods for semi-automated diagnosis (“Accuracy of the estimated non-linear elastic modulus for diagnosing cancer invasiveness” Section).

### Accuracy of measuring cell area changes

We compared the automatically measured area changes with the manually calculated ones. For manual annotations, we drew cell boundaries and segmented photomicrographs into cell areas and backgrounds. Then, we counted the number of pixels in the cell areas. The area change on a pressure level $P_n$ was calculated by dividing the number of pixels on $P_n$ by $P_0 = 0.00~\mathrm{MPa}$.

All 40 cancer cells were used to train and validate the conventional CNN model for measuring changes in cell areas according to pressure levels. The CNN model was trained to predict the manually measured area changes by analyzing photomicrographs captured on $P_n$ and $P_0$. We used 80% of the cancer cells as training data, and the remaining as testing data. By employing 5-fold cross-validation, all cells were used as testing data at least once. Since we captured photomicrographs on six pressure levels (0.00, 0.23, 0.43, 0.63, 0.82, and $1.00~\mathrm{MPa}$), the experiments were conducted with 240 photomicrographs. Becausse of the limited number of cancer cells, we applied data augmentation to cell photomicrographs using ImageDataGenerator in Keras. ImageDataGenerator generates randomly augmented cell images with rotating, horizontal/vertical flipping, zooming, and horizontal/vertical shifting. For batch sizes ($\beta$) and epochs ($\varepsilon$), we conducted a grid search in the ranges of $\beta \in [1,32]$ and $\varepsilon \in [100,800]$ with step sizes $\times 2$ and $+ 100$, respectively. Based on the MAE, $\beta$ and $\varepsilon$ were set as 8 and 200, respectively.

**Figure 9:**
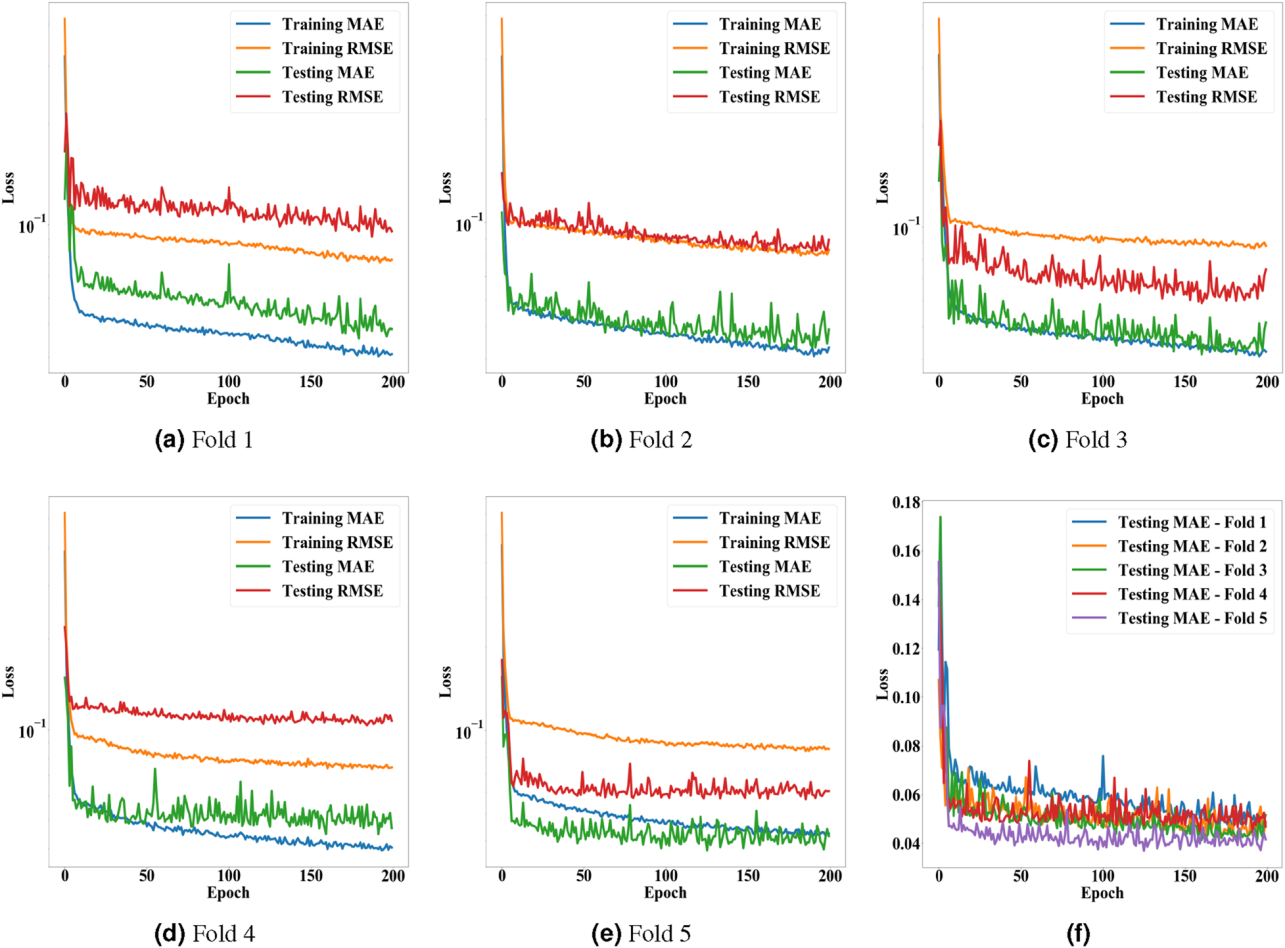
Accuracy of the proposed model for measuring changes in cell areas according to epochs. (**a**–**e**) present the MAE and RMSE of the proposed model on training and testing data from the first to fifth folds. (**f**) shows a comparison of the five folds with each other in terms of MAE.

**Table 1:** Performance of the proposed model for measuring changes in cell areas.

	MAE—training	RMSE—training	MAE-testing	RMSE—testing
Fold 1	0.042	0.079	0.045	0.095
Fold 2	0.044	0.084	0.043	0.084
Fold 3	0.043	0.089	0.041	0.059
Fold 4	0.040	0.075	0.045	0.106
Fold 5	0.046	0.090	0.037	0.059
Average	0.043	0.083	0.042	0.081
S.D.	0.002	0.006	0.003	0.019

This table presents arithmetic means and standard deviations of MAE and RMSE obtained from the 5-fold cross-validation. The left two columns are the results for the training data, and the right two columns are for the testing data.

Figure 9 and Table 1 present the MAE and RMSE of the proposed CNN model on each fold, and the model exhibited stable and high accuracy. The experimental results show that the proposed model can replace the manual approach wherein operators need to draw cell boundaries pixel-by-pixel. In our dataset, the area changes were in the range of [1, 1.81]. Thus, the MAE and its variance of the proposed model are significantly low, considering the range. Even the proposed model achieved lower MAE and RMSE on the testing data than on the training data, despite slightly higher variances. This point and the cross-validation underpin the fact that the proposed model can measure changes in cell area by analyzing photomicrographs without over-fitting. Figure 2 compares area change ratios of the two types of cancer cells (MDA-MB-231 and MCF-7) predicted by the proposed model with the manually measured ones.

### Accuracy of estimating non-linear elastic modulus

Our ground-truth dataset for Young’s modulus consists of only bead data. This section concentrates on presenting how accurately we can approximate $E(c_i) = f(\Delta A(c_i))$. Then, the practicality of the proposed model for real cells is discussed in the next section by classifying cancer cells according to the predicted non-linear elastic modulus automatically.

**Figure 10:**
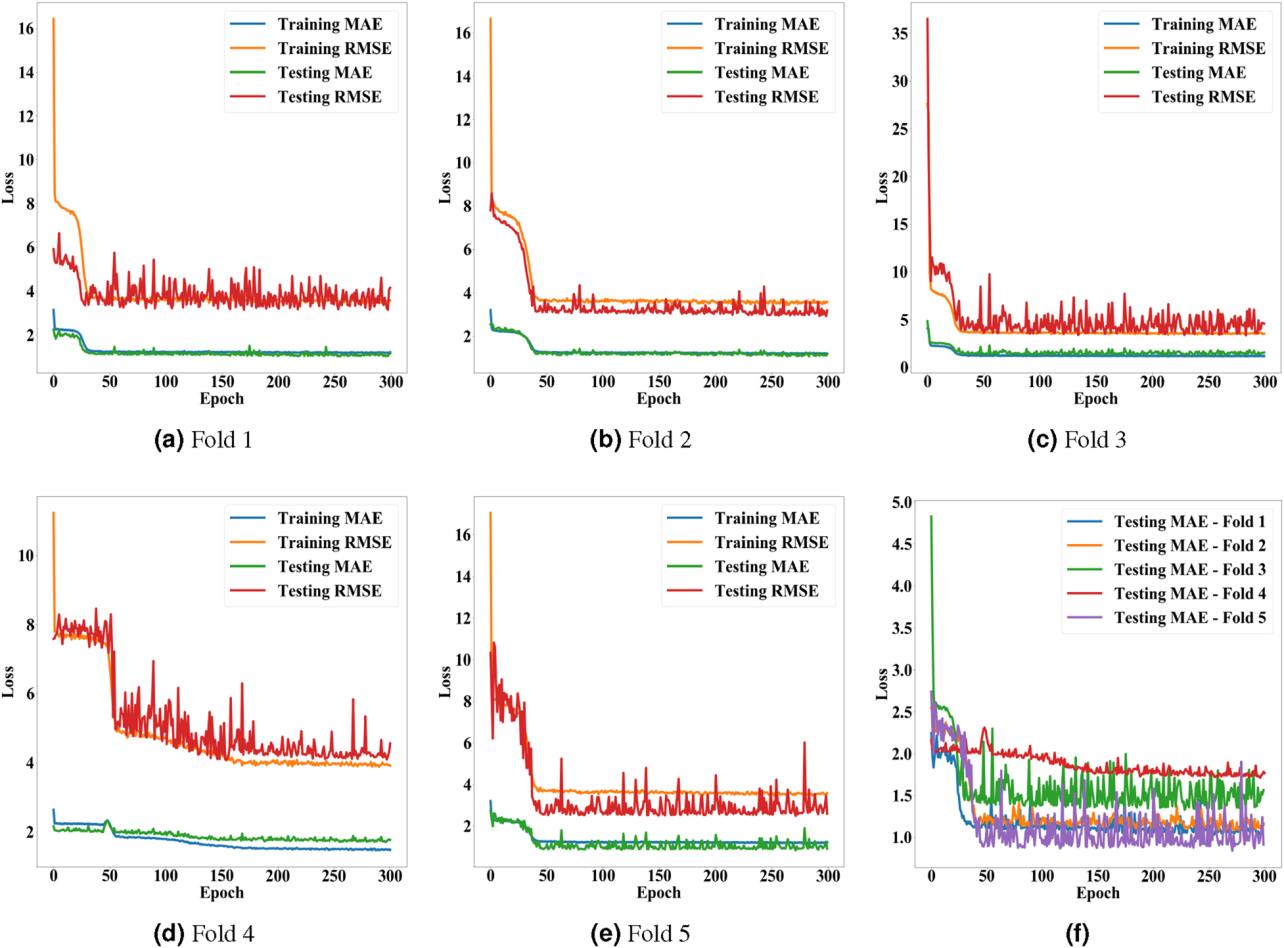
Accuracy of the proposed method for estimating non-linear elastic moduli according to epochs. (**a**–**e**) present the MAE and RMSE of the proposed model on the training and testing data from the 1st to 5th folds. (**f**) shows a comparison of the five folds with each other in terms of MAE.

A total of 100 beads were used to train and test the conventional MLP model for estimating the Young’s moduli of the beads by analyzing their area changes according to pressure levels. The area change ratios of beads were manually measured by counting pixels on photomicrographs. With the 5-fold cross-validation, 80% of the beads were training data, the others were testing data, and all beads were used to validate the model at least once. For batch sizes ($\beta$) and epochs ($\varepsilon$), we conducted a grid search in the ranges of $\beta \in [1,32]$ and $\varepsilon \in [100,800]$ with step sizes $\times 2$ and $+ 100$, respectively. Based on the MAE, $\beta$ and $\varepsilon$ were set as 8 and 400, respectively. Figure 10 presents the accuracy of the proposed model according to the epochs.

**Table 2:** Accuracy of the proposed method for estimating non-linear elastic moduli of beads.

	MAE—training	RMSE—training	MAE-testing	RMSE—testing
Fold 1	1.201	1.788	1.029	1.786
Fold 2	1.211	1.760	1.074	1.609
Fold 3	1.180	1.755	1.323	1.887
Fold 4	1.205	1.759	1.114	1.882
Fold 5	1.200	1.781	0.841	1.424
Average	1.199	1.769	1.076	1.718
S.D.	0.010	0.013	0.155	0.178

This table presents arithmetic means and standard deviations of MAE and RMSE obtained from the 5-fold cross-validation. The left two columns are the results for the training data, and the right two columns are the ones for the testing data.

**Figure 11:**
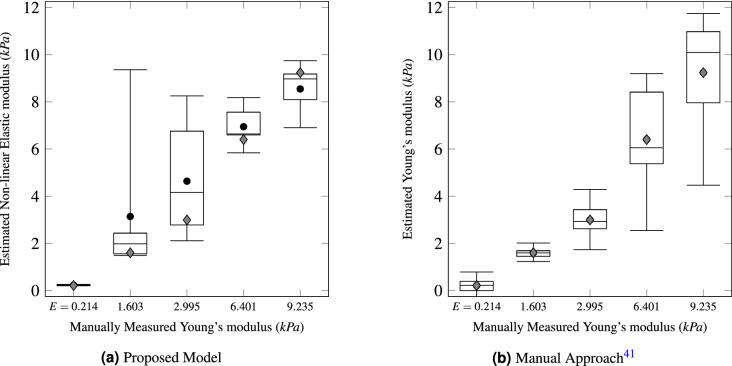
Distributions of the estimated elastic moduli of beads. (**a**) presents the distributions of non-linear elastic moduli of beads estimated by the proposed method, and (**b**) shows the distributions of Young’s moduli of beads predicted by the existing method. The X-axes indicate the manually measured Young’s moduli of the five types of beads, and the Y-axes refer to the estimated elastic moduli of 20 beads for each bead type. The three horizontal lines indicate the first, second, and third quartiles of the estimated elastic moduli, respectively. The tops and bottoms of the whiskers refer to the maxima and minima of the elastic moduli, respectively. Circular dots denote the average elastic moduli. Diamonds mark ground truths.

As shown in Figure 10, the proposed MLP model converged on every fold and achieved stable accuracy. Table 2 presents the average and variance of MAE and RMSE that were obtained from the 5-fold cross-validation. These results indicate that a reasonable accuracy of the proposed model did not come from cherry-picking and can be generalized to other data. Further, we compared the Young’s moduli of beads approximated by the proposed method with those measured by the existing method^41^. This method manually sets the average of the predicted Young’s moduli for a type of bead as the actual Young’s moduli of the beads. Although we can calculate its MAE and RMSE (0.872 and 1.333), it is difficult to achieve fair comparisons for these metrics. Therefore, we compared the distributions of Young’s moduli predicted by the proposed model with those of the existing manual analysis, as displayed in Fig. 11. This comparison shows that the proposed method has a higher resolution for a certain range of Young’s modulus than the other one.

The manual approach estimates Young’s moduli of cells (or beads) by comparing the area change ratios of cells (or beads) with the average area change ratios of beads with particular Young’s moduli. As shown in Fig. 11b, this approach works well on beads with low Young’s moduli. However, in the cases of $E= 6.401~\mathrm{kPa}$ and $E= 9.235~\mathrm{kPa}$, the outputs of the manual approach had high variance, and the ranges of estimated Young’s moduli for the two types of beads overlapped. In other words, beads with low Young’s moduli had more consistent area changes than beads with high Young’s moduli. The proposed model significantly improved the resolution of low deformability (high *E* values), as shown in Fig. 11a. This result indicates that the MLP model found some patterns in the inconsistent area changes of beads with high *E* values. However, the proposed model exhibited considerably higher variances on $E= 1.603~\mathrm{kPa}$ and $E= 2.995~\mathrm{kPa}$ than the manual method. Learning two different area change patterns could hinder accuracy for the border area (i.e., beads with middle *E* values).

### Accuracy of the estimated non-linear elastic modulus for diagnosing cancer invasiveness

This section validates whether the non-linear elastic modulus is useful for diagnosing diseases. We classified cancer cells into invasive and noninvasive groups by using their estimated non-linear elastic moduli with a threshold. The effectiveness of cell deformability for diagnosing cancer invasiveness has been validated by existing studies^41,46^. Thus, the accuracy of the classification results can indirectly underpin whether the proposed method and non-linear elastic modulus are capable of assessing the deformability of live cells. The classification accuracy was measured by precision, recall, accuracy, and $F_1$ measure. Further, we compared the accuracy of the proposed method with the accuracy of the manual method^41^. Because the manual method works based on ground truths, it is not appropriate to directly compare the manual method with the proposed method. However, we use it to show what needs to identify and reveal the inherent deviation of cell data. Also, we did not compare the proposed method with our previous study for deformation-based cell-type classifier^46^. The proposed method and the manual method^41^ aim to estimate elastic moduli of live cells. In these studies, the cell classification is only to evaluate their effectiveness indirectly by examining whether two types of cells have distinguishable elastic moduli since there have not been reliable methods for measuring live cells’ elastic moduli. Therefore, it is difficult to conduct a fair comparison of these elastic modulus estimators with the cell-type classifier (also distant from validating our research questions), although both of them are based on cell deformation analysis. Also, it is worthwhile to note that the predicted non-linear elastic moduli using our proposed method are comparable to that of existing literature, measured by other techniques such as AFM and optical tweezers^4,6–8^. More specifically, existing studies reported that Young’s modulus of MDA-MB-231 and MCF-7 cells was in the range of $1.0 \sim 55.6 ~\mathrm{kPa}$ and $2.8 \sim 87.3 ~\mathrm{kPa}$, respectively, and our proposed method estimated MDA-MB-231 cells as $6.5 ~\mathrm{kPa}$ and MCF-7 cells as $7.2 ~\mathrm{kPa}$.

The cancer cell classification was merely conducted by threshold $\theta _I$. We searched for the optimal $\theta _I$ in $[\min _{\forall c_i} E(c_i),\max _{\forall c_i} E(c_i)]$ with a step size of $+0.05$. According to $\theta _I$, we composed a set of invasive cancer cells $C_I^* = \{ c_i |E(c_i) > \theta _I \}$ and assessed the set using the $F_1$ measure. This can be formulated as:

10 $$\begin{aligned} \theta _I^{*} = {\mathop {\mathrm{argmax}}\limits _{\theta _{I}}} F_1(\theta _{I}), \end{aligned}$$

where $F_1(\theta _I)$ indicates the $F_1$ measure for detecting invasive cancer cells with $\theta _I$. Including the $F_1$ measure, the assessment metrics were calculated as:

11 $$\begin{aligned} P = \frac{|C_I^* \cap C_I |}{|C_I^* |},~ R = \frac{|C_I^* \cap C_I |}{|C_I |},~ F_1 = 2\frac{P \times R}{P + R}, \end{aligned}$$

where $C_I^*$ and $C_I$ refer to sets of predicted and real invasive cancer cells, respectively, and *C* denotes all cells in our dataset.

Both the proposed and existing methods consist of two parts: measuring cell area change ratios and estimating elastic moduli. To validate the effectiveness of each part and the entire system, we compared the following four combinations.

- *Proposed System (SBAT + CNN + MLP)* Using cell photomicrographs with particular pressure levels using SBAT, measuring changes in cell areas by applying the CNN to the photomicrographs, and approximating elastic moduli of cells by applying the MLP to area changes.
- *Manual Approach (SBAT + Manual + Manual)*^41^ Changes in cell areas were measured by counting the number of pixels in the photomicrographs and estimating elastic moduli by using the average gradients of the area change ratios.
- *SBAT + CNN + Manual* Applying the CNN to measuring cell area changes and estimating elastic moduli using the average gradients.
- *SBAT + Manual + MLP* Manually measuring cell area changes and approximating elastic moduli with the MLP.

Table 3 presents the accuracy of the four cases for diagnosing the invasiveness of the cancer cells automatically.

**Table 3:** Accuracy of the four cases for discriminating the invasiveness of cancer cells.

	Proposed model (CNN + MLP)	CNN + Manual	Manual + MLP	Manual Approach^41^(Manual + Manual)
Precision	0.70	0.63	0.75	0.83
Recall	0.70	0.60	0.75	1.00
$F_1$ Measure	0.70	0.62	0.75	0.91

The manual approach works based on ground truths, and it is difficult to directly compare it with the proposed methods. We present its accuracy to demonstrate the goal that we need to identify and reveal the inherent deviation of cell data.

This study employed the MLP model because the area change ratios of cells are not directly proportional to the pressure levels and have high variances, as shown in Fig. 2. Further, the existing method^41^ exhibited high variance on beads with high Young’s moduli. The proposed method improved this point; however, it exhibits higher variance on beads with middle Young’s moduli, as shown in Fig. 11. A comparison of the Manual + MLP case with the manual approach^41^ shows whether the MLP model contributes to distinguishing invasive cancer cells from noninvasive ones. The performance decrement caused by the MLP was significant. The conventional MLP could not perfectly learn the non-linear correlations of area change ratios according to pressure levels with deformability. The manual approach, which ignores the non-linearity by averaging gradients of area changes, achieved high accuracy. This indicates that our dataset was not sufficiently large and diverse to allow the MLP to learn the non-linear correlations.

However, a comparison of the CNN + MLP case with the CNN + Manual case showed that the MLP outperformed the average gradient-based method when used with the CNN. The results of the CNN may result in uncertainty in the inputs of the MLP-based and average gradient-based elastic modulus estimation methods; the outputs of the CNN cannot be more accurate than the ground truths (i.e., manually measured cell area changes).

Thus, the MLP model is more robust against uncertainty in cell area change ratios than the manual method. Similarly, there was a dramatic performance decrement between the CNN + Manual and Manual + Manual cases. Considering that the CNN + MLP and Manual + MLP cases exhibited similar performances, the decrement might come from the same reason: the average gradient-based method was not sufficiently robust against handling uncertainties in CNN outputs.

By comparing the CNN + MLP case with the Manual + MLP case, we note that the proposed CNN model can automate processes for measuring cell area changes with a reasonable performance decrement. The CNN + MLP and Manual + MLP cases achieved $F_1$ measures of 0.70 and 0.75, respectively. The 5 percent point decrement is affordable considering the labor-intensiveness of drawing cell boundaries and counting the number of pixels.

Finally, the manual approach^41^ could not achieve perfect accuracy although it works based on ground truths. This result may be attributed to the diversity of cells (as shown in Fig. 2) and the uncertainty from SBAT operators (as displayed in Fig. 11b). Cells in the same cell line have various deformabilities, and the area change ratios for the same bead are also varied (as shown in Fig. 6). Although we expected that the proposed system would resolve these uncertainties, our dataset was not sufficiently diverse to train the system.

In conclusion, the conventional CNN could automate the cell area change measurement. Because the existing method manually detects cell boundaries and counts the number of pixels in the boundaries, the proposed method contributes to improving the labor-intensiveness of this task. However, the conventional MLP was insufficient to analyze correlations of the cell area change ratios according to pressure levels with the deformability of cells (non-linear elastic modulus).

## Conclusion

This study aimed to automatically measure the non-linear elastic moduli of cells by combining SBAT with conventional neural network models. The SBAT enabled us to deform cells without damaging them. Using the SBAT, we captured photomicrographs of cells by inflicting certain pressure levels on them. The CNN model could automatically measure the degrees of changes in cell areas on photomicrographs captured using the SBAT. Finally, the MLP model could automatically estimate the non-linear elastic moduli of cells by analyzing the cell area changes according to the pressure levels. The proposed method measured the cells’ elastic moduli by learning correlations between Young’s moduli of beads, their deformation, and inflicted pressures. However, this method focused on handling non-linear correlations of cell deformation with pressures and high variance of the deformation. Therefore, we called the estimated degrees of cell elasticity ‘non-linear elastic moduli,’ not Young’s moduli.

The experimental results showed that the proposed system could (semi-) automate the non-linear elastic modulus measurement of live cells. Although our system could not outperform the manual procedures^41^, we significantly reduced the labor-intensiveness in the cell deformability analysis.  “Accuracy of measuring cell area changes and Accuracy of estimating non-linear elastic modulus” Sections showed that the CNN and MLP achieved high accuracy in measuring cell area change ratios and approximating the Young’s moduli of the beads, respectively. In “Accuracy of the estimated non-linear elastic modulus for diagnosing cancer invasiveness” Section, the proposed system exhibited its practicality in diagnosing the invasiveness of breast cancer cells.

Although its accuracy did not outperform that of manual procedures^41^, the proposed system can provide meaningful initial data to clinicians because it is not easy to recognize cell deformability by observing photomicrographs with naked eyes. This study showed the following limitations, and we will focus on resolving these limitations in further research.

- *Inaccuracy of the MLP-based regression* This study attempted to estimate the non-linear elastic moduli of cells by analyzing the correlations of cell area change ratios with pressure levels. However, the MLP model could not outperform the existing method that merely assumes the average gradient of the area change ratios is proportional to Young’s modulus. This failure to outperform the existing method can be attributed to two causes: the conventional MLP was too simple to emulate the correlations, and our data were not diverse enough to reveal the correlations. In further research, we will collect area change data from more beads under different pressure levels and apply recurrent neural networks, which specialize in sequential data, to analyze the area changes according to the pressures.
- *Scale and diversity of datasets* Although the data scale did not create problems in terms of training the models, 40 cells and a disease used in “Accuracy of the Estimated Non-linear Elastic Modulus for Diagnosing Cancer Invasiveness” Section are insufficient to validate the practicality of the proposed system. In the future, we will examine whether the proposed system can (semi-) automate diagnosis of other kinds of disease with a sufficient scale data.
- *Accumulation of uncertainty* In this study, we could not design our system as an end-to-end trainable neural network because it is difficult to measure the Young’s moduli of live cells. Thus, although each part of our system achieved high accuracy, the performance of the entire system had scope for improvement. Our future studies will focus on developing a unified neural network that can measure the non-linear elastic moduli of cells by analyzing the sequences of cell photomicrographs.

## Data Availability

The datasets used and/or analyzed during the current study are available from the corresponding author on reasonable request.
